# Single-molecule imaging of Tau reveals how phosphorylation affects its movement and confinement in living cells

**DOI:** 10.1186/s13041-024-01078-6

**Published:** 2024-02-12

**Authors:** Pranesh Padmanabhan, Andrew Kneynsberg, Esteban Cruz, Adam Briner, Jürgen Götz

**Affiliations:** Clem Jones Centre for Ageing Dementia Research, Queensland Brain Institute, The University of Queensland, 4072 Brisbane, Australia

**Keywords:** Tau, Super-resolution microscopy, Phosphorylation, Single-molecule imaging, Microtubules, Tauopathies, Alzheimer’s disease

## Abstract

Tau is a microtubule-associated protein that is regulated by post-translational modifications. The most studied of these modifications is phosphorylation, which affects Tau’s aggregation and loss- and gain-of-functions, including the interaction with microtubules, in Alzheimer’s disease and primary tauopathies. However, little is known about how Tau’s phosphorylation state affects its dynamics and organisation at the single-molecule level. Here, using quantitative single-molecule localisation microscopy, we examined how mimicking or abrogating phosphorylation at 14 disease-associated serine and threonine residues through mutagenesis influences the behaviour of Tau in live Neuro-2a cells. We observed that both pseudohyperphosphorylated Tau (Tau^E14^) and phosphorylation-deficient Tau (Tau^A14^) exhibit a heterogeneous mobility pattern near the plasma membrane. Notably, we found that the mobility of Tau^E14^ molecules was higher than wild-type Tau molecules, while Tau^A14^ molecules displayed lower mobility. Moreover, Tau^A14^ was organised in a filament-like structure resembling cytoskeletal filaments, within which Tau^A14^ exhibited spatial and kinetic heterogeneity. Our study provides a direct visualisation of how the phosphorylation state of Tau affects its spatial and temporal organisation, presumably reflecting the phosphorylation-dependent changes in the interactions between Tau and its partners. We suggest that alterations in Tau dynamics resulting from aberrant changes in phosphorylation could be a critical step in its pathological dysregulation.

**Supplementary Information:**

The online version contains supplementary material available at 10.1186/s13041-024-01078-6.

Aberrant changes in the phosphorylation state of the protein Tau characterise a range of neurodegenerative disorders, including Alzheimer’s disease and frontotemporal dementia. This hyperphosphorylation is linked to an impairment of neuronal function and eventually, neurodegeneration. Mutagenesis studies have provided valuable insights into the effect of Tau phosphorylation on its subcellular localisation, as well as its spreading, release and aggregation in a disease context [1–4]. For instance, pseudohyperphosphorylation of Tau has been shown to decrease the protein’s ability to interact with microtubules [2], drive its enrichment in dendritic spines [1, 5], increase its release at the plasma membrane through unconventional secretion pathway [6], and alter the location of the axon initial segment, thereby impairing neuronal excitability [3]. However, how the phosphorylation of Tau affects its behaviour at the single-molecule level is only poorly understood.

To investigate this, we transiently expressed wild-type, phosphomimetic and phosphodeficient forms of the prominent 0N4R isoform of human Tau in murine neuroblastoma Neuro-2a (N2a) cells. Single-molecule imaging was performed using the photoactivatable fluorescent protein mEos3.2, which was fused to the carboxy terminus of Tau. Specifically, we replaced 14 disease-relevant serine and threonine residues with glutamic acid (Tau^E14^-mEos3.2) to mimic hyperphosphorylation, or alanine (Tau^A14^-mEos3.2) to abrogate phosphorylation [1, 3, 5] (Fig. 1A). We then used single-particle tracking photoactivated localisation microscopy (sptPALM) [7] and total internal reflection fluorescence (TIRF) illumination (Fig. S1A) [8] to localise and track individual wild-type (Tau^WT^-mEos3.2) and mutated Tau molecules near the cytosolic leaflet of the plasma membrane with ~ 100 nm axial resolution (Fig. S1B and S1C). (For more details, see Materials and Methods in Additional File 1.) In this region, Tau assumes its various functions by interacting with its multiple partners: lipids in the plasma membrane, membrane-associated proteins, including the kinase Fyn and the calcium-regulated membrane-binding protein annexin A2, cytoskeletal elements, including actin filaments and microtubules, and motor proteins, such as dynactin, a co-factor for the cytoplasmic microtubule motor protein dynein-1 [9]. The diffraction-limited TIRF images captured before single-molecule imaging revealed more prominent microtubule filament-like structures in Tau^A14^-mEos3.2-expressing cells compared to those expressing Tau^E14^-mEos3.2 (Fig. 1B, C and Fig. S2). This observation possibly reflects biochemical findings showing a reduction in the association of Tau with microtubules as a result of phosphorylation at these 14 sites [2].

**Fig. 1:**
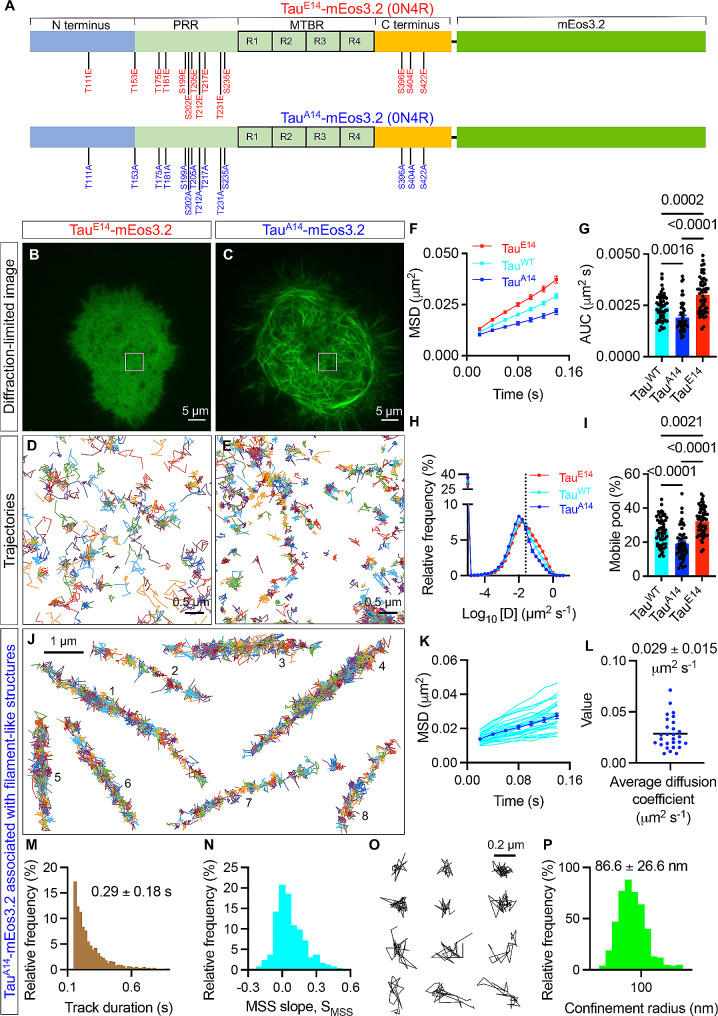
Tau phosphorylation at 14 residues increases the mobility of Tau near the plasma membrane. A, Schematic of 0N4R Tau tagged with mEos3.2 at the C-terminus with 14 critical Ser/Thr residues replaced by glutamic acid (Tau^E14^-mEos3.2) or alanine (Tau^A14^-mEos3.2). B, C, Representative diffraction-limited TIRF image of N2a cells expressing Tau^E14^-mEos3.2 (B) or Tau^A14^-mEos3.2 (C) acquired in the green channel before sptPALM imaging. D, E, Maps of trajectories of Tau^E14^-mEos3.2 and Tau^A14^-mEos3.2 molecules corresponding to the boxed region shown in panels B and C, respectively. F-I, Comparison of Tau^WT^-mEos3.2, Tau^E14^-mEos3.2 and Tau^A14^-mEos3.2 mobility parameters in live N2a cells. F, G, Average MSD as a function of time (F) and the corresponding area under the curve (AUC) (G). H, J, Distribution of the diffusion coefficients and the corresponding fraction of mobile pool (I). The dashed line indicates the threshold (Log_10_ [D] ≤ − 1.6) demarcating the immobile and the mobile pools of Tau molecules. Error bars, s.e.m. J, Examples of trajectories of Tau^A14^-mEos3.2 molecules associated with filament-like structures. K, L, Average MSD of Tau^A14^-mEos3.2 trajectories associated with filament-like structures (K) and the corresponding average diffusion coefficient (L). M, Distribution of duration of Tau^A14^-mEos3.2 tracks associated with filament-like structures. N, Distribution of the MSS slope, S_MSS_, of individual Tau^A14^-mEos3.2 tracks associated with filament-like structures. O, Examples of Tau^A14^-mEos3.2 trajectories associated with filament-like structures classified as immobile or confined pools by MSS analysis. P, Distribution of the confinement radius of immobile and confined pools of Tau^A14^-mEos3.2 molecules from filament-like structures. N = 56 cells expressing Tau^WT^-mEos3.2, 49 cells expressing Tau^A14^-mEos3.2, and 55 cells expressing Tau^E14^-mEos3.2 from 5 independent experiments each. In G and I, statistical analysis was performed using one-way ANOVA with the Tukey’s multiple comparison correction

Next, we recorded a time series of detections of either Tau^WT^-mEos3.2, Tau^E14^-mEos3.2 or Tau^A14^-mEos3.2 in the red emission channel (561 nm excitation) at 50 Hz for a duration of 160 s. This allowed us to readily construct thousands of Tau^WT^-mEos3.2, Tau^E14^-mEos3.2 and Tau^A14^-mEos3.2 trajectories per cell (Fig. 1D, E). We first performed a moment scaling spectrum (MSS) analysis [10] to characterise the mobility pattern of the mutated Tau molecules. As recently observed for Tau^WT^-mEos3.2 [8], both Tau^E14^-mEos3.2 and Tau^A14^-mEos3.2 molecules exhibited a heterogeneous mobility pattern, existing in immobile, confined, and freely diffusive states near the plasma membrane (Fig. S3). We then calculated the average mean square displacement (MSD) and the frequency distribution of the instantaneous diffusion coefficient of all trajectories from each cell (Fig. 1F-I and Fig. S4). The slope of the average MSD curve (Fig. S4) and the area under the average MSD curve (AUC) (Fig. 1F, G) were larger for Tau^E14^-mEos3.2 molecules and smaller for Tau^A14^-mEos3.2 molecules compared to Tau^WT^-mEos3.2 molecules. Moreover, the diffusion coefficient distribution of Tau^E14^-mEos3.2 molecules shifted towards higher values, and the mobile fraction of Tau^E14^-mEos3.2 molecules was significantly higher than that of Tau^WT^-mEos3.2 and Tau^A14^-mEos3.2 molecules (Fig. 1H, I). These observations indicate that Tau^E14^-mEos3.2 molecules are more mobile and Tau^A14^-mEos3.2 molecules are less mobile than wild-type Tau near the plasma membrane in live cells. Notably, we previously found that phosphomimetic mutations at positions 262 and 356 (12E8 epitope) in the microtubule-binding domain, distinct from the 14 mutations that are outside of the microtubule-binding domain and were examined in this study, also increased Tau mobility in N2a cells [8].

In the high-resolution intensity maps of Tau^A14^-mEos3.2 (Fig. S5), we observed signatures of filament-like structures that were also visible in the diffraction-limited TIRF images (Fig. S2) and resembled the well-studied microtubule architecture. We extracted Tau^A14^-mEos3.2 trajectories associated with each of these structures (Fig. 1J and S6) and computed the average MSD curve (Fig. 1K) and the average diffusion coefficient, *D*_*avg*_ (Fig. 1L). We found that the *D*_*avg*_ values varied substantially across the filament-like structures (Fig. 1L) and that the duration of these Tau^A14^-mEos3.2 trajectories was 0.29 ± 0.18 s (mean ± s.d.) (Fig. 1M). Moreover, the MSS analysis (Fig. 1N, O) identified individual Tau^A14^-mEos3.2 trajectories associated with these structures that were immobile and confined, and the confinement radius of the immobile and confined Tau^A14^-mEos3.2 trajectories was 86.6 ± 26.6 nm (mean ± s.d.) (Fig. 1P).

In conclusion, our sptPALM approach demonstrated that both Tau^E14^ and Tau^A14^ molecules are detectable and exhibit heterogeneous mobility patterns close to the cytosolic leaflet of the plasma membrane of N2a cells. Given that the likelihood of tracking free cytosolic Tau molecules under TIRF illumination is low due to their high diffusion coefficient values [11], the observed Tau^E14^ and Tau^A14^ trajectories are likely to represent molecules that are interacting with the components of the plasma membrane and its associated cytoskeleton. Our results reveal that the mobility of Tau^E14^ molecules is significantly higher than that of Tau^A14^ molecules. This is probably due to a combination of two factors: the release of Tau^E14^ molecules from the microtubules and an increased binding and trapping of Tau^A14^ molecules on the microtubules. Recent studies have shown that Tau undergoes unconventional secretion through the plasma membrane in neuroblastoma cells and neurons, and that the Tau^E14^ mutant is released to a larger degree than the Tau^A14^ mutant in SH-SY5Y neuroblastoma cells [2, 6]. Whether the increased mobility of Tau^E14^ molecules observed in our study is causally linked to increased Tau^E14^ secretion remains to be tested. The filament-like structures observed in cells expressing Tau^A14^-mEos3.2 presumably represent Tau^A14^ molecules associated with microtubules. The duration (~ 0.29 s) of the Tau^A14^-mEos3.2 trajectories associated with the filament-like structures observed in our study is much longer than the reported dwell time (40 ms) of wild-type 2N4R Tau on microtubules in live PC12 cells [12]. It is plausible that the higher affinity of Tau^A14^ towards microtubules could lead to repeated binding and release of Tau^A14^ on microtubules, resulting in a longer track duration. Overall, our super-resolution-based approach combined with those of others [2, 12–15] offers a powerful framework to examine the impact of physiological and pathological modifications to Tau on its organisation, behaviour and function in different cellular compartments.

### Electronic supplementary material

Below is the link to the electronic supplementary material.

Supplementary Material 1

## Data Availability

All data associated with this study are available within the manuscript and its Additional Files.

## Abbreviations

sptPALM: Single-particle tracking photoactivated localisation microscopy
TIRF: Total internal reflection fluorescence
MSS: Moment scaling spectrum
MSD: Mean square displacement
AUC: Area under the curve
D_avg_: Average diffusion coefficient
