# Supplementary material for: Single-molecule imaging of Tau reveals how phosphorylation affects its movement and confinement in living cells
Source: Mol Brain. 2024 Feb 12;17:7. doi: 10.1186/s13041-024-01078-6 (PMC10863257; doi:10.1186/s13041-024-01078-6)
Supplement: Supplementary file 1 — Supplementary Material 1 [file 13041_2024_1078_MOESM1_ESM.docx]

**Single-molecule imaging of Tau reveals how phosphorylation affects its movement and confinement in living cells**

Pranesh Padmanabhan^1,*^, Andrew Kneynsberg^1^, Esteban Cruz^1^, Adam Briner^1^ and Jürgen Götz^1,*^

^1^Clem Jones Centre for Ageing Dementia Research, Queensland Brain Institute, The University of Queensland, Brisbane, Australia 4072

**MATERIALS AND METHODS**

**Cell culture, plasmids, and transfection**

As described previously [1], murine Neuro-2a (N2a) cells were maintained in Dulbecco’s minimum essential medium (ThermoFisher; Catalog No. 11965-092) supplemented with 10% fetal bovine serum (Bovogen; Catalog No. SFBS-F) and 50 U/ml penicillin/streptomycin (Gibco; Catalog No. 15140-122). Cells were cultured at 37°C under 5% CO_2_, and 250,000 cells per well were seeded onto 12-well plates and allowed to adhere for 18 h before transfection.

Mammalian expression plasmids were made using human tau cDNA for isoform 0N4R and the human cytomegalovirus promoter. Fusion proteins were created with a C-terminal tag of mEos3.2 (RRID: Addgene #54550) [2]. Tau^E14^-mEos3.2 and Tau^A14^-mEos3.2 constructs were generated by mutating residues T111, T153, T175, T181, S199, S202, T205, T212, T217, T231, S235, S396, S404, and S422 to glutamic acid (E14) or alanine (A14), respectively.

For transfection, we used Lipofectamine^TM^ LTX and Plus reagent (Invitrogen; Catalog No. 15338030). For single-molecule imaging, the cells were dissociated with trypsin-EDTA (0.25%; Catalog No. 25200-056), centrifuged, and ~32,000 cells replated onto 35 mm glass-bottom culture dishes (Ibidi) coated with poly-D-lysine for 1-2 h. The medium was then replaced, and cells were imaged after ~23 to 56 h.

**Single-molecule imaging and analysis**

We performed live-cell TIRF microscopy-based imaging using an iLas^2^ azimuthal TIRF illumination system (Roper Scientific) mounted on a Nikon Ti-E inverted microscope, with a 100x/1.49 NA oil-immersion TIRF objective (CFI Apochromat, Nikon) and Evolve 512 Delta EMCCD cameras (Teledyne Photometrics). MetaMorph (version 7.10.1.161, Molecular Devices) was used to perform single-molecule imaging of mutated Tau tagged with mEos3.2 at 50 Hz for a duration of 160 s at 37 °C. Cells were washed and incubated with buffer A (145 mM NaCl, 5 mM KCl, 1.2 mM Na_2_HPO_4_, 10 mM D-glucose, 20 mM HEPES, pH 7.4) during imaging. To perform sptPALM, we used a 405 nm laser (Stradus 405, Vortran Laser Technology) to photoconvert the mEos3.2-tagged molecules, a 561 nm laser (Cobolt Jive, Cobolt Lasers) for excitation of the photoconverted molecules, a TIRFM GFP/RFP filter cube (Nikon Corporation) in the microscope body, a T565lpxr long-pass dichroic beam splitter, and an ET600/50m emission filter (Chroma Technology) in the TwinCam (Cairn Research) dual emission image splitter transmission arm. The 405 nm laser power density was between 3.4 x 10^-6^ and 9.5 x 10^-5^ kW/cm^2^, and the 561 nm laser power density was set to ~0.14 kW/cm^2^.

We localised and tracked individual mutated Tau molecules tagged with mEos3.2 with the PALM-Tracer tool that operates as a plugin of Metamorph software (Molecular Devices) and using a frame-to-frame particle-linking distance threshold of 318 nm (3 pixels). Intensity and trajectory maps were constructed, mean square displacement and diffusion coefficients were computed, and moment scaling spectrum analysis [3] was performed as described previously [1]. GraphPad Prism 10.0.1 was used to perform statistical tests.

**Figure S1. Single-molecule imaging of mutated Tau near the plasma membrane of N2a cells. A,** Schematic of detection of mutant Tau molecules fused with mEos3.2 tag in a TIRF microscopy setup. **B,** Two examples of detection of individual Tau^E14^-mEos3.2 in consecutive frames. The time interval between each frame is 20 ms. Scale, 500 nm. **C,** Representative trajectories of the Tau^E14^-mEos3.2 molecule shown in panel B. Scale, 200 nm.

**Figure S2. More filament-like structures were observed in cells expressing Tau^A14^-mEos3.2 have. A, B,** Low-resolution TIRF images of live cells expressing either Tau^E14^-mEos3.2 (A) or Tau^A14^-mEos3.2 (B) acquired in the green channel.

**Figure S3. Tau^A14^-mEos3.2 and Tau^E14^-mEos3.2 molecules exhibit heterogeneous mobility patterns.** **A, B,** A gallery of trajectories of Tau^A14^-mEos3.2 (A) and Tau^E14^-mEos3.2 (B) molecules displaying immobile (top row), confined (middle row) and diffusive (bottom row) motion states inferred using moment scaling spectrum analysis.

**Figure S4. The average diffusion coefficient of Tau^E14^-mEos3.2 and Tau^A14^-mEos3.2.** **A,** The average MSD of trajectories from a representative cell expressing Tau^E14^-mEos3.2 was fitted (4 points) by the equation *MSD*(*τ*) = a + 4*D_avg_τ*, where *D_avg_* is the average diffusion coefficient, *τ* is the time lag and *a* is the y-intercept. **B,** Estimates of the average diffusion coefficient values of Tau^WT^-mEos3.2, Tau^E14^-mEos3.2 and Tau^A14^-mEos3.2 corresponding to data in Fig 1F. Statistical analysis was performed using one-way ANOVA with the Tukey’s multiple comparison correction.

**Figure S5. Intensity maps of Tau^A14^-mEos3.2.** Single**-**molecule localisation of Tau^A14^-mEos3.2 molecules colour-coded based on their local density. Regions of interest from four different cells are shown. The local density of each detection was determined by computing the number of detections within a circle of 30 nm radius**.**

**Figure S6. Tau^A14^-mEos3.2 trajectories associated with filament-like structures.** In each example, all the trajectories are in cyan in the left panel, and Tau^A14^-mEos3.2 trajectories associated with filament-like structures used for analysis are highlighted in blue in the right panel.

**References**

1. Padmanabhan P, Kneynsberg A, Cruz E, Amor R, Sibarita JB, Götz J: **Single-molecule imaging reveals Tau trapping at nanometer-sized dynamic hot spots near the plasma membrane that persists after microtubule perturbation and cholesterol depletion**. *EMBO J* 2022, **41**:e111265.

2. Zhang M, Chang H, Zhang Y, Yu J, Wu L, Ji W, Chen J, Liu B, Lu J, Liu Y *et al*: **Rational design of true monomeric and bright photoactivatable fluorescent proteins**. *Nat Methods* 2012, **9**:727-729.

3. Vega AR, Freeman SA, Grinstein S, Jaqaman K: **Multistep track segmentation and motion classification for transient mobility analysis**. *Biophys J* 2018, **114**:1018-1025.
